# SHORT syndrome in two Chinese girls: A case report and review of the literature

**DOI:** 10.1002/mgg3.1385

**Published:** 2020-06-29

**Authors:** Yanhong Zhang, Baolan Ji, Jinsheng Li, Yanying Li, Mei Zhang, Bo Ban

**Affiliations:** ^1^ Department of Endocrinology, Affiliated Hospital of Jining Medical University Jining Medical University Jining China; ^2^ Chinese Research Center for Behavior Medicine in Growth and Development Jining China; ^3^ Department of Endocrinology Henan Hongli Hospital Changyuan City China

**Keywords:** diabetes, insulin resistance, *PIK3R1*, short stature, SHORT syndrome, whole exome sequencing

## Abstract

**Background:**

SHORT syndrome is a rare inherited multisystem disease that includes characteristic facial features, growth retardation, and metabolic anomalies and is related to heterozygous mutations in the *PIK3R1* gene. However, it is difficult to ascertain the relationship between the phenotype and the genotype quickly and efficiently.

**Methods:**

We report two Chinese girls with SHORT syndrome who presented with growth retardation, dysmorphic features, insulin resistance, and diabetes. Comprehensive medical evaluations were collected, including anthropometric measurements, laboratory measurements, and imaging examinations. Whole exome and Sanger sequencing was performed to detect and confirm the underlying genetic mutations in these patients. We prescribed metformin for the patients.

**Results:**

The patients both presented diabetes, insulin resistance, short stature, lipodystrophy, and characteristic facial dysmorphic features. A heterozygous mutation was detected in the *PIK3R1* gene (c.1615_1617del) of Patient 1. The analysis of patient 2 revealed another *PIK3R1* mutation (c.1945C>T). After family validation, neither their parents nor their brothers had similar clinical presentations or carried the same mutation.

**Conclusion:**

We identified two de novo heterozygous mutations in *PIK3R1* as the cause of SHORT syndrome in two Chinese girls. Additionally, in terms of diabetes control, metformin works well in the early treatment stage.

## Graphical abstract

SHORT syndrome is a rare inherited multisystem disease that includes characteristic facial features, growth retardation and metabolic anomalies and is related to heterozygous mutations in the PIK3R1 gene. To date, ten different mutations in the PIK3R1 gene have been reported. After searching the literature, we found that there were correlations between genotype in PIK3R1 and the phenotype of SHORT syndrome to a certain extent.

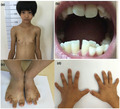

## 1 INTRODUCTION

SHORT syndrome (OMIM #269880) is a rare autosomal dominant condition of multiple anomalies whose features are summarized by short stature, hyperextensibility of joints, ocular depression, Rieger anomaly, and teething delay (Chung & Gibson, 2014) along with mild intrauterine growth restriction, partial lipodystrophy, delayed bone age, hernias, and progeroid appearance, which are additional features of the disease (Anthony, Christopher, & Robert, 1989; Rainer, Leticail, & Sigrun, 2003; Reardon & Temple, 2008). Most patients show normal intelligence (Rainer et al., 2003).

The morbidity and mortality of this disease are unclear. To date, fewer than 50 cases have been reported in the literature (Magali et al., 2016). SHORT syndrome is associated with multiple mutations in the *PIK3R1* gene (5q13.1), which encodes phosphatidylinositol 3‐kinase regulatory subunit alpha. Mutations in this gene are testified to impair the *PI3K/AKT/mTOR* pathway, which plays an important role in cellular proliferation and growth. Several related research teams have independently reported the finding of mutations in *PIK3R1* as the primary cause of SHORT syndrome (Chudasama et al., 2013; Dyment et al., 2013; Schroeder et al., 2014; Thauvin‐Robinet et al., 2013). To date, there have been ~10 mutations evidently associated with SHORT syndrome, such as c.1929_1933delTGGCA, 1945C>T, c.1615_1617delATT, c.1465G>A, c.1943dupT, and c.1892G>A, which are related to insulin resistance and lipodystrophy. Rieger anomaly has been observed in patients with mutations in c.1906_1907insC as well as in patients with c.1971T>G and c.1945C>T.

Clinical criteria for the diagnosis and treatment of SHORT syndrome have not yet been determined. Given that the disease can cause dysfunction in multiple systems and adversely affect the health of patients and the next generation, further research is needed. In this study, we report two Chinese girls presenting with complicated clinical manifestations who were finally diagnosed with SHORT syndrome due to a mutation in the *PIK3R1* gene. We have also reviewed the literature about this rare syndrome to provide a clear picture of this syndrome.

## 2 CASE PRESENTATION

### 2.1 Patient 1

Patient 1 was a 14.5‐year‐old girl born to a primigravida from Shandong Province. At conception, her father was 31 years old, and her mother was 30 years old. She was delivered in good general condition, without asphyxia or hypoxia. She was born at term but displayed features of intrauterine growth restriction (IUGR) during pregnancy. Her birth weight was 2100 g (<3 percentile), and her height was 44 cm (<3rd percentile). From day 3 on, the baby was exclusively artificially fed until 2 years old, which resulted in gradual low weight gain. Her psychomotor development was normal. She started to practice speaking at 12 months but walked at 19 months. At school age, she found it difficult to learn, especially in math, and the score was usually less than 20 (out of 100 points). Short stature was noted at birth but became more noticeable from the age of 12 years; however, her parents did not record the growth rate per year in detail. She was first seen in the endocrine clinic at age 14.5 years and was referred for the assessment of short stature.

**Figure 1:**
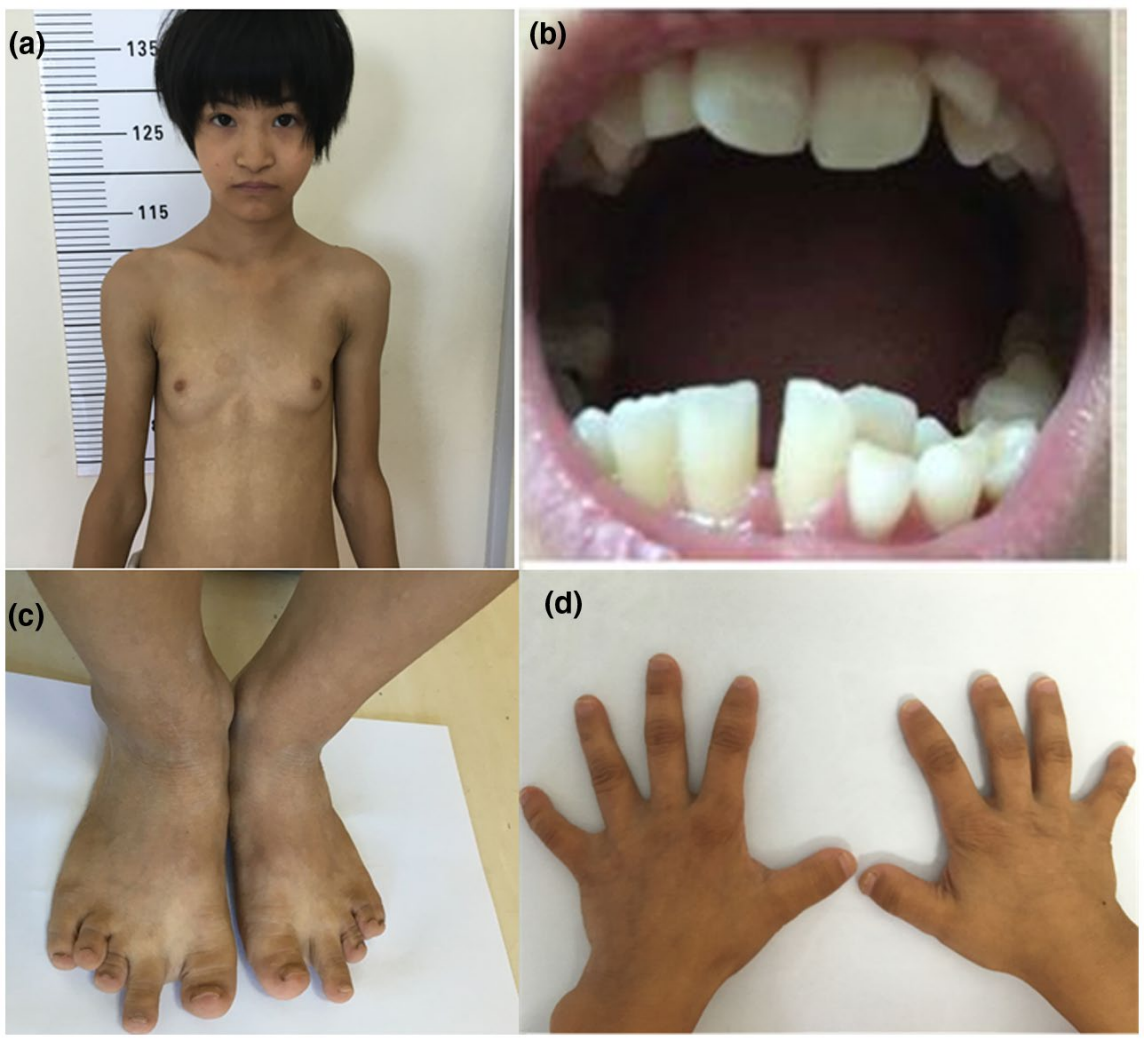
Facial appearance and other typical features of Patient 1. (a) General appearance, (b) mixed dentition, (c) short 3rd and 4th toes of the feet, (d) short 4th and 5th metacarpals of the hand

At admission, we performed a systematic physical examination. She was noted to have partial lipodystrophy as well as a peculiar face, looking considerably older than her age. On examination, she was found to have short stature (137.3 cm, −4.0 *SD*), low weight (27 kg, −2.7 *SD*), and low BMI (14.3 kg/m^2^, −2.5 *SD*). Her face showed typical features, such as microcephaly, triangular shape, small chin, and so on. Physical examination showed abnormal abduction of the elbow joint (elbow valgus) and brachydactyly, short fingers/toes, metacarpal sign and bilateral toe deformities (Figure 1). Her puberty stage was Tanner III but no menarche.

Her father was 170 cm in height and grew significantly at the age of 14 years. Her mother was 153 cm in height with menarche at the age of 13 years. Her younger brother was 6 years old and maintained a height above the 50th percentile of the population with the same age and gender. There was no family history of diabetes mellitus or short stature.

### 2.2 Patient 2

Patient 2 was the second child of nonconsanguineous parents, aged 28 and 27 years, who were from Henan Province. She was born in the 32nd week of gestation. Her birth weight was 2000 g (<50 percentile), but her birth length was not available. Because of difficulty with sucking, she was gavage fed for several months. Her teething was delayed, and she had malocclusion. At the age of 1 year, she underwent surgery for left‐sided inguinal hernia. She started to walk alone at 18 months and spoke her first words at 2 years old. Her height and weight gain were so slow that she seemed to be languidly weak and thin during her childhood. She attended regular school at the age of 7 and showed less than average academic performance. Menarche was at 14 years, and menses were regular since that time. The girl inadvertently noticed a darker urine color 1 day and underwent a urine test at a local outpatient clinic. The urinary sugar was 4+, which tends to indicate diabetes, so she came to our endocrine clinic for help.

**Figure 2:**
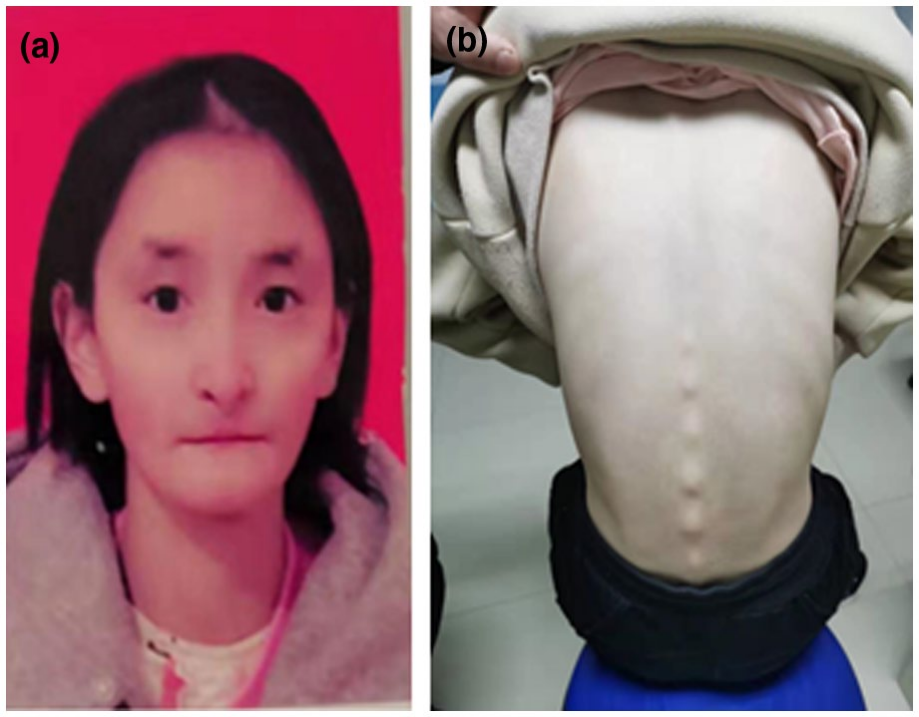
Facial appearance and other typical features of Patient 2. (a) Facial dysmorphic signs included a triangular shape of the face, micrognathia, deep‐set eyes, hypoplastic alae nasi and low‐hanging columella, (b) subcutaneous fat was reduced, especially on the chest and back

On examination at age 16, she was severely underweight (35 kg, −2.1 *SD*). Her height was just slightly above the third percentile (152 cm, −1.5 *SD*). Facial dysmorphic signs included a triangular shape of the face, micrognathia, deep‐set eyes, hypoplastic alae nasi, and low‐hanging columella (Figure 2a). Subcutaneous fat was reduced, especially on the chest and back (Figure 2b). Her puberty stage was Tanner V.

Her father is 172 cm, grew significantly at the age 15 years. Her mother is 158 cm and had menarche at age 14 years. Her elder brother is 22 years old and his height is 180 cm. There is no family history of diabetes mellitus or short stature.

## 3 METHODS

### 3.1 Editorial policies and ethical considerations

This study was approved by the Ethics Committee of the Affiliated Hospital of Jining Medical University and Henan Hongli Hospital (China). The study also follows the principles outlined in the Helsinki Declaration and the parents of these two patients gave written informed consent for molecular study and publication.

### 3.2 Anthropometric measurements

Anthropometric measurements included measurements of height, weight, systolic blood pressure (SBP), diastolic blood pressure (DBP), and pubertal stage. Height and weight were assessed according to standard procedures with the participants in light clothing, with no shoes. Body height was measured to the nearest 0.1 cm using a Best Industrial Stadiometer (Nantong Best Industrial Co, Ltd.). A scale with a capacity of 120 kg (Wuxi Weigher Factory Co, Ltd.) was used to measure body weight to the nearest 0.1 kg. Height and weight were expressed as the standard deviation scores (SDS) based on normative values for Chinese children (Li, Ji, Zong, & Zhang, 2009a). BMI was calculated as weight divided by height in metres squared, and the BMI SDS was calculated according to 2009 growth charts for Chinese children and adolescents (Li, Ji, Zong, & Zhang, 2009b). Pubertal stage was evaluated by physical examination according to the Tanner stages (Wright et al., 2012).

### 3.3 Laboratory measurements and imaging examination

GH was measured by the chemiluminescence method (ACCESS2, Beckman Coulter) with an analytical sensitivity of 0.01 µg/L. Serum IGF‐1 and IGFBP‐3 concentrations were estimated based on a chemiluminescence assay (DPC IMMULITE 1000 analyzer; SIEMENS) with an intra‐assay and inter‐assay coefficient of variation of 3.0 and 6.2%, respectively. Total cholesterol (TC), high‐density lipoprotein cholesterol (HDL‐C), LDL‐C, very‐low‐density lipoprotein cholesterol (VLDL‐C), triglyceride (TG), alanine aminotransferase (ALT), fasting plasma glucose (FPG), and uric acid (UA) levels were determined using an auto‐biochemical analyzer (Cobas c702, Roche; Shanghai, China). Measurements of thyroid function (including free T3 [FT3], free T4 [FT4], thyroid‐stimulating hormone [TSH]), gonadotropin, cortisol rhythm and adrenocorticotropic hormone (ACTH) were tested by a luminescence immunoassay system (Cobase 602; Roche). HbA1c was determined by high‐performance liquid chromatography (ADAMS HA‐8180). C peptide and insulin were tested by an electrochemiluminescence analyzer (Cobas e801; Roche), and antibodies against GAD and ICA were determined by enzyme‐linked immunosorbent assay (Thermo Multiskan FC). Bone age assessment was obtained by the interpretation of a left hand X‐ray image by the Greulin‐Pyle Atlas method.

### 3.4 Genetic testing

Genomic DNA was isolated from the blood of the patients, parents, and brothers as previously described (Kang et al., 2019). The whole exome was captured by the SeqCap EZ MedExome Target Enrichment Kit (Roche) and was sequenced by WuXi NextCODE Gene Sequencing Technology Service Co., Ltd. The sequence was read by Illumina HiSeq 2500 (Illumina). The read length of the paired end was 150 bp, and the average coverage of the capture area was ~100×. The sequencing results were compared with the human genome reference sequence (UCSC GRCh38.p12), and multiple database (OMIM, ESP, Clinvar, 1000 Genomes HGMD) annotations were used to locate candidate genes and mutations. The full *PIK3R1* gene DNA sequence (NM_181523.2) was downloaded from the NCBI website. Finally, we performed Sanger sequencing verification of candidate home DNA samples.

## 4 RESULTS

### 4.1 The comparison of typical features of SHORT syndrome with these two patients

As shown in the above figure, the patients presented the most common signs and symptoms of SHORT syndrome except for Rieger abnormality. The comparison of features typical of SHORT syndrome in these two patients is shown in Table 1.

**Table 1:** The comparison of typical features of SHORT syndrome with these two patients

Typical clinical features	SHORT syndrome	Patient 1	Patient 2
SHORT acronym signs	**S** (short stature) <−2 *SD*	+	–
	**H**yperextensibility of joints/inguinal **H**ernia	+	+
	**O** (ocular depression)	–	+
	**R** (**R**ieger abnormality)	–	–
	**T** (**T**eething delay)	+	+
Facial dysmorphism	Triangular face	+	+
	Prominent forehead	–	–
	Hypoplastic or thin alea nasi	+	+
	Mild midface hypoplasia	–	–
	Small chin or micrognatia	+	+
	Thin lip and downturned mouth	+	+
	Large low‐set ears	+	+
	Progeroid face	–	–
Other signs	Anterior chamber of eye abnormalities	–	–
	Lipoatrophy	+	+
	Thin, wrinkled skin and visible veins	–	–
	Glaucoma	–	–
	Insulin resistance	+	+
	Diabetes	+	+
	Ovarian cysts	–	–
	Intellectual deficiency	+	–
	Speech delay	+	+

Abbreviations: −, absence of a feature; +, presence of a feature.

### 4.2 The clinical metabolic features of the patients

In our patients, most of the biochemical examinations were normal, such as liver function, kidney function, lipids, and electrolytes. However, Patient 1 showed severe diabetes and insulin resistance, with a fasting plasma glucose of 15.3 mmol/L, glycated hemoglobin of 12.5%, blood ketone body quantification of 4.7 mg/dl, urine ketone 2+, and fasting C peptide of 4.12 ng/ml. IGF‐1 was 644 µg/L, and IGFBP‐3 was 10.1 mg/L, which were all within the normal range. The antibodies for diabetes were all negative. Peripheral blood chromosomes showed 46,XX. Pituitary magnetic resonance imaging (MRI) showed normal structures. In terms of imaging examination, the bone age was 15 years old. The antibodies for ICA and GAD were all normal. Patient 2 showed a similar metabolic profile, as presented in Table 2.

**Table 2:** The clinical biochemical and hormonal tests of these two patients

Typical clinical features	Patient 1	Patient 2
Gender	Female	Female
Weight at birth (g)	2100	2000
Height at birth (cm)	44	na
Age at assessment (years)	14.5	16
Bone age (years old)	15	17
Height at assessment (cm)	137.3	152
Weight at assessment (kg)	27	35
BMI at assessment (kg/m^2^)	14.3	15.1
Fasting plasma glucose (nmol/l)	15.3	14.4
HbA1c (%)	12.5	13.1
C peptide (ng/ml)	4.12	2.86
Insulin (µIU/ml)	na	26.13
IGF‐1 (µg/L)	644	na
IGFBP‐3 (mg/L)	10.1	na
Triglycerides (mmol/L)	0.93	0.92
Total cholesterol (mmol/L)	3.92	4.4
Free T3 (pmol/L)	5.11	5.88
Free T4 (pmol/L)	19.95	9.77
TSH (mIU/L)	1.21	2.63
E (pg/ml)	36.98	<20
LH (mIU/ml)	16.75	17.03
25‐(OH) VD (ng/ml)	20.2	21.26

Abbreviation: na, no available.

### 4.3 Pathogenic *PIK3R1* mutations in patients with SHORT syndrome

A heterozygous mutation was detected in the *PIK3R1* gene (c.1615_1617del), which led to the deletion of an amino acid (Ile539del) in the frame of the protein encoded by the *PIK3R1* gene in patient 1.

In patient 2, we detected the transition of C>T at position c.1945 of the *PIK3R1* gene (c.1945C>T, p.Arg649Trp). After family validation, their parents and brothers did not have similar clinical presentations or the same mutation site. Neither parent carried the mutation.

### 4.4 Treatment and follow‐up

At the beginning, Patient 1 was treated with insulin aspart by continuous subcutaneous insulin infusion (CSII), while Patient 2 with insulin lispro. Initially, the patients showed little response to insulin treatment, even the daily insulin dose increased to 76 Units. Finally, we discontinued the insulin analog and switched to metformin 250 mg three times a day to Patient 1 and Patient 2, and then, the concentration of plasma glycaemia gradually decreased to 6.0 and 6.46 mmol/L, respectively, after 1 month of therapy. However, in Patient 2, the insulin level increased to 50.04 µIU/ml, which was higher than before.

## 5 DISCUSSION

As more patients are identified through genetic testing, it is increasingly clear that the original name SHORT syndrome does not fully describe the complete phenotypic spectrum of this syndrome. To date, fewer than 50 cases have been reported in the literature. Avila presented a meta‐analysis of the available clinical data of SHORT syndrome patients before 2016 (Magali et al., 2016). Nearly 80% of patients presented with short stature, small chin, and midface hypoplasia; more than 90% presented with small chin and low‐set ears; and nearly 100% presented with ocular depression, teething delay, triangular face, and deep‐set eyes. However, less than 50% of patients presented with the hyperextensibility of joints and Rieger sign.

The clinical features of the patients reported here comprise short stature, partial lipoatrophy, microcephaly, facial gestalt, and delayed teething, which are in accordance with previously reported features of SHORT syndrome. In addition, Patient 1 had metacarpal signs and bilateral toe deformities. To date, no similar abnormalities have been reported. The metacarpal signs and bilateral toe deformities may be a variable part of this syndrome or a manifestation of a multifactoral trait expressed coincidently.

SHORT syndrome is caused by mutations in the *PIK3R1* gene (5q13.1), encoding phosphatidylinositol 3‐kinase regulatory subunit p85α, which plays an important role in chemical signal transduction within cells, including cell growth and proliferation, protein synthesis, and the maturation of adipocytes. Mutations in this gene have been shown to impair the *PI3K/AKT/mTOR* pathway. Related research teams have independently reported the finding of mutations in *PIK3R1* as the primary cause of SHORT syndrome (Chudasama et al., 2013; Dyment et al., 2013; Schroeder et al., 2014; Thauvin‐Robinet et al., 2013). To date, 10 different mutations in the *PIK3R1* gene have been reported as the underlying cause of SHORT syndrome (Table 3). The most common mutation site in the affected individuals is the Arg649Trp missense mutation. Other missense mutations (Glu489Lys, Arg631Gln), as well as a deletion (Ile539del), truncation (Tyr657*), and frame‐shift insertions (Asn636Thrfs*18, Asp643Aspfs*8, Arg649Profs*5), have also been described (Bárcena et al., 2014; Klatka, Rysz, Kozyra, Polak, & Kołłątaj, 2017). In the present study, we found a de novo heterozygous mutation in the *PIK3R1* gene (c.1615‐1617del; p. IIe539del) in Patient 1, which has been reported to be related to insulin resistance and lipoatrophy (Thauvin‐Robinet et al., 2013). In patient 2, we detected the transition of C>T at position c.1945 of the *PIK3R1* gene (c.1945C>T, p. Arg649Trp), which tends to be the hotspot mutation site.

**Table 3:** Pathogenic *PIK3R1* mutations and related phenotype in SHORT syndrome

DNA nucleotide change	Protein amino acid change	Related phenotype	References
c.1465G>A	p.Glu489Lys	S, O, R, T, IR, partial lipoatrophy	Thauvin‐Robinet et al. (2013)
c.1615_1617delATT	p.Ile539del	S, O, T, IR, partial lipoatrophy	Thauvin‐Robinet et al. (2013)
c.1892G>A	p.Arg631Gln	S, IR, partial lipoatrophy	Thauvin‐Robinet et al. (2013)
c.1906_1907delAA	p.Asn636ProfsTer17	S, R	Klatka et al. (2017)
c.1906_1907insC	p.Asn636Thrfs*18	S, R, T, IR, partial lipoatrophy	Dyment et al. (2013)
c.1929_1933delTGGCA	p.Asp643Aspfs*8	S, H, O, R, IR, partial lipoatrophy	Bárcena et al. (2014)
c.1943dupT	p.Arg649ProfsTer5	S, O, partial lipoatrophy	Thauvin‐Robinet et al. (2013)
c.1945C>T	p.Arg649Trp	S, O, R, T, lipoatrophy	Chudasama et al. (2013)
c.1971T>G	p.Tyr657Ter	S, H, O, T, partial lipoatrophy	Thauvin‐Robinet et al. (2013)
c.1956dupT	p.Lys653	S, O, partial lipoatrophy	Klatka et al. (2017)

Abbreviations: H, hyperextensibility of joints; IR, insulin resistance; O, ocular depression; R, Rieger anomaly; S, Short stature; T, teething delay.

After searching the literature, we also found that there were correlations between genotype in *PIK3R1* and the phenotype of SHORT syndrome to a certain extent. Most of the patients had typical facial anomalies, such as triangular shape, prominent forehead, small chin and aged appearance (Chudasama et al., 2013; Dyment et al., 2013; Schroeder et al., 2014; Thauvin‐Robinet et al., 2013). There were also some relationships between certain genotypes and metabolic problems in these patients. For example, mutations such as c.1929_1933delTGGCA, c.1945C>T, c.1615_1617delATT, c.1465G>A, c.1943dupT and c.1892G>A were related to insulin resistance and lipodystrophy. Rieger anomaly has been observed in patients with c.1906_1907insC, c.1971T>G and c.1945C>T mutations. In our study, the patients both presented insulin resistance and lipodystrophy with mutations in the *PIK3R1* gene (c.1615‐1617del, c.1945C>T) but without Rieger anomaly. We should strengthen follow‐up to observe the abovementioned changes in the patients.

Multisystem involvement is a feature of SHORT syndrome, and short stature and diabetes are found in over half of patients. Clinical criteria for the treatment of SHORT syndrome have not yet been determined. However, it is highly feasible to offer comprehensive treatment for metabolic abnormalities over time.

Individuals with SHORT syndrome are usually born at or slightly before term and typically exhibit mild intrauterine growth retardation (IUGR); varying degrees of short stature are usually present throughout childhood. As reported in the literature, adult height in male patients with SHORT syndrome was between 155 and 163 cm, and in females, it was between 143 and 160 cm. As previously reported, one patient was treated with recombinant human growth hormone for 2 years in Verge's study (Verge, Donaghue, Williams, Cowell, & Silink, 1994). The pretreatment growth rate was 4.9 cm/year, increasing to 11.3 cm/year during the first year of therapy and 8.8 cm during the second year. After 25 months of growth hormone therapy, the patient presented with diabetes. The rhGH therapy can induce hyperinsulinaemia, which might aggravate insulin resistance and accelerate the onset of diabetes in subjects with SHORT syndrome. Therefore, we should not use growth hormone to treat short stature in Patient 1. We provided some helpful advice, such as improving nutrition, increasing exercise and ensuring adequate sleep.

In addition to the typical appearance, the patients in this article had prominent metabolic abnormalities, such as insulin resistance and diabetes. Initially, the patients showed little response to insulin treatment. Finally, we prescribed metformin to Patient 1 and Patient 2, and then, the concentration of plasma glycaemia gradually decreased to 6.0 and 6.46 mmol/L, respectively, after 1 month of therapy. However, in Patient 2, the insulin level increased to 50.04 µIU/ml, which was higher than before the treatment. Some researchers found that metformin might worsen rather than improve insulin resistance and glucose tolerance (Lewandowski, Dąbrowska, Brzozowska, Kawalec, & Lewiński, 2019). The precise cause of such profound and paradoxical worsening of glucose tolerance after metformin treatment remains unknown and requires the analysis of more SHORT syndrome cases and long‐term follow‐up of the patients.

The prognosis of SHORT syndrome is related to age, the number of involved organs, the function of the involved organs, and the response to treatment. For the most part, life expectancy is unaffected. As an autosomal dominant disease, there is a 50% chance of transmission to the next generation; therefore, genetic counseling should be performed in these patients.

In conclusion, we found a de novo heterozygous mutation in the *PIK3R1* gene (c.1615‐1617del; c.1945C>T). Our patients presented the most common clinical features of SHORT syndrome, such as short stature, lipoatrophy, insulin resistance and diabetes. We prescribed metformin to them, and then, the concentration of plasma glycaemia gradually decreased, but insulin resistance seemed more obvious. Familiarization and discernment of dysmorphic features is of great significance in the diagnosis of complicated genetic syndromes and enables early implementation of appropriate management and proper care of the patient.

## CONFLICTS OF INTEREST

The authors declare no conflict of interest.

## AUTHOR’S CONTRIBUTIONS

Yanhong Zhang and Baolan Ji contributed equally to this work and are co‐first authors. Yanhong Zhang carried out the studies and drafted the manuscript. Baolan Ji revised the manuscript carefully. Jinsheng Li participated in data collection of Patient 2. Yanying Li participated in data colletion of Patient 1. Mei Zhang and Bo Ban participated in the design of the study also revising it critically for important intellectual content and final approval of the version to be published. All authors read and approved the final manuscript.

## Data Availability

The datasets that support the findings of this study are available from the corresponding author upon reasonable request.
